# 

**Published:** 1911-01-07

**Authors:** 


### Appointments and Resignations.

Mr. T. Arnold Johnston, M.D., Ch.B. Edin., has been appointed honorary pathologist to the Leicester Infirmary for a period of one year.

The following changes have taken place in the resident staff of the Leicester Infirmary: Mr. C. A. Joll, M.B., F.R.C.S., has been appointed senior house surgeon; Mr. A. D. Child, M.B., Ch.B. Edin., second house surgeon; Mr. J. H. Bennett, L.R.C.P., assistant house physician; and Mr. M. M. Morrison, M.B., Ch.B. Edin., and Mr. A. Semple, M.B., Ch.B. Glas., assistant house surgeons, the latter to March 31, 1911.

### Obituary.

The death is reported at Vienna of Mr. Edward Howard Collens, of Hereford, aged thirty-six. He studied at Mason College, Birmingham, took his diploma in 1893, and then became M.B. of London University in the following year. He had been house surgeon to the Queen's Hospital, Birmingham, and was surgeon to the Hereford City Police.

The death is reported, at Geelong, Victoria, Australia, of Mr. Stephen Maberly Smith, at the age of fifty-six. Mr. Smith took his M.R.C.S. in 1875, and his L.R.C.P. (Edin.) in 1876, in which year he went to Dublin, and, after studying at the Rotunda Hospital there, obtained his L.M. A bicycle accident, succeeded by pneumonia, was the cause of his death.

The death is announced of Mr. Edgar Richard Senhouse Lipscomb, formerly of Starcross, Devon, at the age of fifty-one. Mr. Lipscomb, who had retired temporarily from practice, and was living at Hove, Sussex, took his M.R.C.S. and L.S.A. in 1883, and his L.R.C.P. in 1885. He was a student at Guy's, and his appointments included that of surgeon to the Western Counties Idiot Asylum, Starcross.

Mr. Edward Arnold Cloete-Smith has died at his residence, Hyde Park. His father was the late Deputy-Surgeon-General C. E. Smith, of the Army Medical Service. He himself studied at St. Bartholomew's Hospital, and obtained the conjoint qualifications in 1884. He lived in Bengal, India, for some years, and devised a method of local anæsthesia for surface operations, and was the inventor of an improved otoscope.

The death has taken place, at Durban, South Africa, after an operation, of Mr. Frank Reginald Henry Potts. Mr. Potts, who was born in 1863, studied at Guy's Hospital and obtained the M.R.C.S., becoming a L.R.C.P. in 1890. He was district surgeon and health officer to the Lower Tugela Division of the Victoria Company, Natal, and served in the war as Surgeon-Captain of the Natal Volunteer Regiment, and was also a civil surgeon in the Royal Army Medical Corps. Before going to South Africa, Mr. Potts' medical appointments in this country included those of medical officer to the Wiltshire County Asylum, clinical assistant to the Evelina Hospital for Children and the Hospital for Consumption, Victoria Park, house surgeon to the Brecon County Hospital, the Wallasey Dispensary, and the Seacombe Hospital, Birkenhead.

News has been received from Paris of the death of Dr. George Joseph Bull. Dr. Bull practised for many years in the Rue de la Paix, and had a classic reputation as an ophthalmic specialist. He had professional friends in England, America, and Canada, and took a prominent part at the annual meeting of the British Medical Association in London in 1895. Dr. Bull, who was born at Hamilton, Ontario, in 1848, obtained his M.D. degree at McGill University, Montreal, in 1869. After practising in New York and at Denver he went to Paris in 1886, and obtained a French degree. He was ophthalmic surgeon, hon. secretary, and treasurer of the Continental Anglo-American Medical Society and ophthalmic surgeon to the Hertford British Hospital and to the American Hospital. Dr. Bull was the inventor of several instruments for examining the eye, as, for instance, Bull's Cross and Bull's ophthalmometer.

### Jottings.

A proposal is under consideration to reconstruct Chester Infirmary as a memorial to King Edward. An anonymous donor has offered to contribute £1,000 provided the work is begun at once. The scheme is estimated to cost from £15,000 to £25,000.

A British hospital is to be established at Monte Video.

Guy's Hospital has been fined 10s. for using armorial bearings without a proper licence.

The fund for extending Croydon General Hospital as a memorial to King Edward, including £500 from Mr. Frank Lloyd, now amounts to £2,529.

King Edward's Hospital Fund for London has received at the Bank of England from the League of Mercy its contribution for the year 1910 of £18,000.

On Thursday, February 16, a lecture on "The Public Control of Hospitals" will be given in the Caxton Hall by Mr. Joseph Clayton, under the auspices of the Humanitarian League.

League of Mercy.—Tuesday, January 10th, Buckinghamshire District. Lady Susan Trueman "At Home," Town Hall, Chesham, 3 p.m. H.H. Princess Victoria of Schleswig-Holstein (Lady President of the District) will preside.

The Norwegian Deaconess Home and Hospital of Chicago dedicated its new addition on November 20. The building measures one hundred and twenty by fifty feet, and is four storeys high. The hospital now has accommodation for one hundred patients.

An entertainment is being given to-day (Saturday) to the poor children attending the out-patient departments of the Royal Free Hospital, Gray's Inn Road. The Carlton Hotel has kindly given a large Christmas-tree and a number of toys, which will be distributed on this occasion.

Seamen's Hospital Society (Dreadnought), Greenwich, S.E.—The quarterly general court of governors of this Corporation will be held at the Institute of Chartered Accountants, Moorgate Place, Moorgate Street, E.C., on Thursday, January 12, 1911, at five o'clock precisely.

H.R.H. Princess Christian will receive gifts this (Saturday) afternoon, on the occasion of the Annual Pound Day, at the Holiday Home, Englefield Green, and will afterwards preside at the Christmas-tree to the inmates, who are cripple boys selected by the Ragged School Union from their register of 7,000 crippled children.

One of the cottages of the State Hospital for the Insane at Toledo, Ohio, was completely destroyed by fire on the night of November 30. No lives were lost, credit being due to the prompt and efficient work of the hospital officers and firemen in quelling the panic which followed the alarm among the two hundred insane patients in the hospital.

The Bootle Borough Hospital, Liverpool, has decided to reconstruct its northern wing, which is out of date and not in keeping with the newer southern wing. The Mayor of the borough has promised his support, and the public will be invited to assist the scheme, as the local memorial to King Edward, which it is hoped will be completed in this, the Coronation year of his son.

### TO HOSPITAL SECRETARIES AND OTHERS.

We invite contributions from any of our readers, and shall be glad to pay, according to length, for items of news for the institutional section (including appointments, resignations, gifts, etc.) which we may publish. All payments for manuscripts used are made as early as possible after the beginning of each quarter.

### Gifts and Bequests.

The Samaritan Free Hospital for Women has received £5 from the Saddlers' Company.

Mrs. Alice Maria Smithson, of Huddersfield, has left £500 to the Dewsbury and District Infirmary.

Lord Winterstoke has given £5,000 to the special fund being raised for the extension of the Bristol General Hospital.

Miss Anne Chapman Douglas, of Forthside, Trinity, Edinburgh, left the residue of her estate to the Leith Hospital.

Mr. William Stuttaford, of Clevelands, Worcester Park, Surrey, a South African merchant, left £100 to the Somerset Hospital, Cape Town.

The Leicester Infirmary has received a donation of £100 from Mr. H. C. Hartley, and a grant of £15 from S. D. R. S. D. (London).

Mr. Whitelaw Reid, the American Ambassador, has given £100 to the Prince Francis of Teck Memorial Fund for the endowment of the Middlesex Hospital.

Prince Alexander of Teck has received from the Mercers' Company £500 towards the Prince Francis of Teck Memorial Fund for the endowment of the Middlesex Hospital.

Mrs. Gulielma Frances Moss, of Boscombe, Hants, and Streatham, has bequeathed the residue of her estate, which will apparently amount to about £15,000, to the Bolingbroke Hospital, Wandsworth Common, S.W.

Mr. Henry Pidcock, of The Hermitage, Worcester, retired solicitor, left £500 to the Worcester Infirmary, £300 to the Royal Albert Orphanage, Worcester, £150 each to the Foundling Hospital, London, Dr. Barnardo's Homes, the Cancer Hospital, Brompton, and the Consumption Hospital, Kensington.

Dr. Charles Wolliscroft Belfield, M.R.C.S., of Brislington House, Brislington, Somerset, who died on November 9, left estate valued at £31,380 gross, with net personalty £29,430. Dr. Belfield left £5,000 to the Bristol General Hospital, and £100 each to the Bristol Eye Hospital and the Bristol Royal Infirmary.

The following donations have been received in aid of the Ancoats Hospital appeal fund : The Wholesale Co-operative Society, Ltd., £100 ; "In Memory" of F. Entwistle, Esq., J.P., £20 ; St. Aidens Presbyterian Church, Didsbury, "Help Guild and friends, £10 8s. ; The Electro Motors, Ltd., donation, £25 ; annual subscription, £5 5s. ; F. Craven Moore, Esq., M.D., F.R.C.P., £21 ; Belsize Motors, Ltd., £5 5s.

Mr. James Lynch, of Ballinclare House, Camolin, co. Wexford, left £300 to St. Michael's Hospital, Kingstown ; £100 each to St. Vincent's Hospital, Dublin, the Mater Misericordiæ Hospital, the Jervis Street Hospital, Dublin, the Children's Hospital, Temple Street, Dublin ; £100 each to the Hospital for Incurables, Donnybrook, and the Sick and Indigent Roomkeepers' Society. The amount available for charitable purposes would appear to be about £15,000.

Mr. John Birkmyre, of Broadstone, Kilmalcolm, Renfrew, formerly managing director of the Gourock Ropework Company, Limited (in which his interests are valued for probate at £187,594), and of the firm of Messrs. Birkmyre Bros., of Calcutta (in which his interest is valued at £108,800), who died on August 9 last, aged seventy-five, left £5,000 to the trustees of the Broadstone Jubilee Hospital, Port Glasgow, of which he was the founder, for the endowment fund.

Mr. John Richmond Tibbald, of Wimborne Road, Bournemouth, has bequeathed £150 to the Royal Victoria Hospital, Bournemouth.

### THE WEEK'S NOVELTIES.

### INSTITUTIONAL, MEDICAL AND GENERAL.

**STAUFFER'S DRY YEAST** tablets and powder are, perhaps, the best form in which to prescribe yeast in those conditions in which this somewhat unpleasant remedy is likely to be efficacious. The preparation thoroughly deserves a trial in cases of constipation. Many patients will find the tablets much more convenient and, be it added, more palatable than the fashionable varieties of sour milk.

**BOVRIL** has had the honour of receiving the Royal Warrant from H.M. the King. It will be remembered that Bovril was favoured with the royal appointment to King Edward VII., and it may not be generally known that at the death of the Sovereign all Royal Warrants expire.

There is more skill required in the making of Surgeons' Needles than is probably generally known. Those manufactured by **W. WOODFIELD AND SONS**, Redditch, are of English make and English quality, and their present popularity among operators is the best guarantee of the quality of their material, and the flexibility and strength of the material from which they are made. Messrs. Woodfield invite inquiries from all interested in specialties or particular details, and will supply any information that the profession and public may wish to make on these points.

The day of the flock mattress is gone, virtually, for ever, the special report of the Local Government Board giving to it probably the final death-blow. That report, revealing the horrors that flock may readily harbour, has made institutions and the public turn naturally to horsehair as an improved and hygienic substitute. This substitute has been offered to the public for some time by the **LANCASHIRE BEDDING COMPANY**, Marlborough Street, Vauxhall Road, Liverpool, which provides a horsehair mattress which, the firm states, can never grow hard or lumpy. As these uninviting qualities are not the peculiarity of horsehair but the penalty of unskilful workmanship, the firm's reputation in institutional circles is enough to guarantee their claim in this respect. The mattress is made up from horsehair which has been thoroughly cleansed, and is sewn in the best British tick. The firm's mattress, measuring 3 feet 6 inches by 6 feet 6 inches, will be packed free by rail to any address in the country for the reasonable price of 17s. 6d.

In these days of drugs and adulteration the profession is only too glad to find some standard of quality for pharmaceutical products, and this can be obtained best by the reputation of a particular firm, which has maintained its position as a purveyor above suspicion. **THE BAYER COMPANY, LTD.**, of 19 St. Dunstan's Hill, London, E.C., has an extensive and well-known list of pharmacons, and its name upon a preparation of that useful but delicate drug veronal, or of heroin hydrochloride, is a testimony of the attempts made to standardise the drug market. The aim of high-class pharmacy to-day must be the standardisation of popular remedies, and that difficult task the above firm of Bayer's has kept in view.

In spite of electricity and the many varied forms under which gas, or some ally of acetylene, loves to disguise itself, the lamp can never be given up as an inevitable form of house light. A good one possesses the qualities of high reflective power, freedom from smell, and the consumption of readily obtainable fuel. These are to be expected from **BRUCE'S PARABOLIC REFLECTOR LAMP**, which, at the modest price of 4s. 6d., has held its own in institutional favour for twelve years. No hospital can afford to be without the finest science and skill can give in the host of appliances necessary for its existence. A lamp that is used in a hospital is a lamp that will give satisfaction anywhere. The illustrated catalogue of F. and J. Bruce, 232 Borough High Street, London, S.E., contains details of all the firm's designs.

[Further particulars of these preparations are given in our advertisement columns this week.]

MR. PIERPONT MORGAN has given $40,000 for the improvement of the hospital at Aix-les-Bains. Dr. Guyenot, the superintendent of the hospital, is now in America for the purpose of studying their hospital methods.

Just Issued. Ninth Edition, Rewritten and Enlarged, 9s. net.

### PHARMACY, MATERIA MEDICA & THERAPEUTICS.

Being a Commentary on the B.P. and its Drugs, with their Preparations, and on all the new Non-official Remedies in use, with Chapters on the different processes used in Dispensing, Prescription-writing, Latin Glossary, &c. By Sir W. WHITLA, M.D., LL.D., Professor, Materia Medica, Queen's University, Belfast, and Senior Physician, Royal Victoria Hospital.

By the same Author. New Work. 1,900 pages. Crown 8vo. 25s. net.

### PRACTICE OF MEDICINE.

Containing in two Handy Volumes a complete and independent Article on the Etiology, Symptoms, and Diagnosis of every Medical Disease; arranged in Dictionary Form for Practitioners and Students.

London: Baillière, Tindall & Cox, 8 Henrietta Street, W.C.

